# Anthocyanins Function as Anti-Inflammatory Agents in a* Drosophila* Model for Adipose Tissue Macrophage Infiltration

**DOI:** 10.1155/2018/6413172

**Published:** 2018-03-12

**Authors:** Alice Valenza, Carola Bonfanti, Maria Enrica Pasini, Paola Bellosta

**Affiliations:** ^1^Department of Biosciences, University of Milano, Via Celoria 26, 20133 Milan, Italy; ^2^Center of Integrated Biology (CiBio), University of Trento, Via Sommarive 9, 38123 Trento, Italy

## Abstract

Epidemiological and preclinical studies have demonstrated that bioactive foods like flavonoids, polyphenolic compounds derived from fruits and vegetables, exert a protective action against obesity, cardiovascular disorders, and Adipocyte Tissue Macrophage infiltration (ATM). All these pathologies are characterized by increase in reactive oxygen species (ROS) and in proinflammatory cytokines that have been shown to favor the migration of immune cells, particularly of macrophages, in metabolically active organs like the liver and adipose tissue, that in* Drosophila *are constituted by a unique organ: the fat body. This study, using a unique* Drosophila* model that mimics human ATM, reveals the beneficial effects of flavonoids to reduce tissue inflammation. Our data show that anthocyanin-rich food reduces the number of hemocytes,* Drosophila* macrophages, infiltrating the fat cells, a process that is associated with reduced production of ROS and reduced activation of the JNK/SAPK p46 stress kinase, suggesting a fundamental function for anthocyanins as antioxidants in chronic inflammation and in metabolic diseases.

## 1. Introduction

Obesity is a metabolic syndrome occurring worldwide and often associated with other chronic diseases such as cardiovascular disorders, type II diabetes, and cancer [1].

The onset of obesity is the result of multifactorial elements, including a sedentary lifestyle, genetic predisposition, ethnicity, and environmental factors (such as organic pollutants) [2]; these factors with a diet rich in fats and sugars and poor in phytonutrients may result in weight gain and subsequently lead to metabolic disorders [3, 4]. Obesity is known to trigger a low-grade inflammation in metabolically active tissues and in organs such as the liver and adipose tissue [5–8]. Inflammation is the result of cellular and humoral responses with the scope to protect the organism from various insults, including infection and tissue damage, in attempt to rescue tissue homeostasis [9, 10].

In humans, the adipose tissue regulates lipid homeostasis and in normal conditions controls the storage of energy reserves in the form of triglycerides as well as functioning as an endocrine organ, producing a variety of proinflammatory cytokines such as IL-1, IL-6, and IL-8, IFN*γ*, and TNF*α* [11, 12]. In pathological conditions, such as obesity or metabolic syndrome, the adipocytes start to alter the production of these proinflammatory cytokines, which results in the activation of the innate immune system with recruitment of immune cells including macrophages leading to a state of chronic inflammation or ATM [7]. In addition, lipid accumulation and chronic inflammation in obese people are associated with a permanent increase of oxidative stress and with the production of high levels of reactive oxygen species (ROS) [13, 14], which is often associated with the activation of the c-Jun-NH_2_-terminal kinase (JNK/SAPK) p46, member of a mitogen-activated protein kinases (MAPKs) downstream of JNK signaling [15]. This pathway is highly conserved in* Drosophila* and consists of a cascade of phosphorylation events starting with the activation of the JNKKK kinases, consisting of the Ask1 and Tak1, that activate MKK7, the orthologue of Hemipterous (Hep), and terminates with the activation of JNK/SAPK p46 kinase, called* basket (bsk)* in Drosophila, that is negatively regulated by* Puckered (puc), *a phosphatase, which itself is a target of JNK/SAPK p46 kinase (see Figure 3(e)) [16].

This pathological situation influences other organs by altering their functions. Furthermore the adipose tissue from obese individuals exhibits a reduced capacity to store fat leading to an increase of circulating free fatty acids (FFAs) that promotes insulin resistance and damages the mitochondrial membrane thereby enhancing the production of ROS causing oxidative stress [17–19].

Epidemiological evidence suggests that a high intake of bioactive food is associated with a lower risk of developing chronic diseases like obesity [20]. Bioactive foods may influence the physiological and cellular activities of oxidative pathways and in recent years the attention has been focused on a class of secondary metabolites present in plant foods called flavonoids that seem to possess beneficial properties in preventing chronic diseases [21, 22]. The possible health benefits of flavonoids are linked to their potent antioxidant and free radical scavenging activities demonstrated* in vitro* and* in vivo* using different animal models [23]. Among the different classes of flavonoids, anthocyanins represent the major red, purple, and violet pigment in many plants and fruits.* In vivo* studies showed that anthocyanins added to the diet stimulate the secretion of insulin and decrease the generation of ROS [21, 24]. Preclinical studies performed on human demonstrate that dietary anthocyanins have a positive biological effect against obesity-induced inflammation and oxidative stress [24, 25], which is associated to a lower risk of type 2 diabetes. This potentially important application creates a high interest in understanding the action of these natural bioproducts in preventing metabolic diseases.

In order to study the mechanisms that control the anti-inflammatory response to flavonoids, we took advantage of a previously unrecognized conserved functional relationship between the immune cells, called hemocytes (macrophage like cells) and adipocytes (larval fat body, FB) [26].* Drosophila *FB, a metabolic tissue with similar physiological functions to the mammalian adipose tissue and liver, acts as a functional unit to control key metabolic processes and the native immune response, in addition to storing fats and sugars [27]. In* Drosophila* the immune response is orchestrated by the hemocytes that are circulating cells in the hemolymph, present at all stages of the life cycle, and compose the fly's innate immune system [9, 28–33]. Hemocytes are essential mediators in the cell-cell communication process: they have been shown to mediate a response between the fat body and tumor cells to control their growth [34] and to promote proliferation of epithelial cells in response to the release of ROS following cell death in cells of the imaginal discs [35].

Using our model of obesity, we observed that hemocytes infiltrate the FB of obese larvae mimicking the chronic inflammation present in human obesity (manuscript in submission). In this study we report that treatment with anthocyanin-enriched food results in a significant decrease in the number of hemocytes infiltrating the FB concomitantly to a reduction in ROS and of the phosphorylation of JNK/SAPK stress kinase.

Our data demonstrate that the mechanisms driving the protective role of bioproducts like anthocyanins* in vivo* as anti-inflammatory and antioxidants are conserved in* Drosophila.* In addition, they highlight the potential use of our model to study the complex relationship between inflammation and obesity and corroborate the positive action of anthocyanins to combat chronic inflammation in humans.

## 2. Materials and Methods

### 2.1. Fly Stocks and Husbandry

Hml-RFP/CyO is a gift from Katja Brückner at UCSF. The P0206-Gal4 from [36], UAS-CG7839RNAi (BL 25992), is an RNA interference lines that reduces the expression of the CG7839 gene encoding for the orthologue of the yeast ribosomal protein Noc1; herein the CG7839-RNAi construct will be called Ni. Fly cultures and crosses were grown on standard fly food composed of yellow corn, sugar, and yeast molasses-base, at 25°C.

*Feeding Experiment and Chemical Compounds. *Crosses were kept in culture bottles perforated to provide adequate air circulation and eggs were collected on a grape agar plate (5%) supplemented with dry yeast every 3 hours. First instar larvae were collected after 24 hours AEL (after egg laying) and shifted into vials containing different food. First instar larvae were reared with 2 g of standard food, hereafter Normal Food (NF) and 5 ml of each flavonoid (FL) extract, one containing only flavonoids (NF + FL) and another extract containing flavonoids and 0.24 mg/ml anthocyanins (NF + FL + ACN). All these phenolic compounds were extracted from the cobs of yellow and purple corn (gift from Katia Petroni and Chiara Tonelli, University of Milan); only the purple extract is rich in anthocyanins, while the content of other flavonoids is the same in both extracts (the content of flavonoids present in the extracts are reported in [37]).

### 2.2. Hemocytes Quantification and Size Analysis in Larval Fat Bodies

To label* in vivo* plasmatocytes, which comprise more than 95% of the hemocytes population in the* Drosophila *larva, we used the transgene* Hml*Δ*-DsRed (Hml-RFP)* that contains the promoter for hemolectin, expressed in the hemocytes, fused with the Red Fluorescence Protein (RFP) [38]. Fat bodies from 20 larvae at 5 and 12 days AEL were dissected in phosphate-buffered saline (PBS) pH 7.4 and fixed in 4% paraformaldehyde (PFA) for 30 minutes. Hoechst 33258 (Sigma Aldrich) was added to stain DNA in a final concentration of 1 *μ*g/ml. After washing with PBS, fat bodies were mounted onto slides with DABCO-Mowiol and images were acquired using an SP2-LEICA Lasertechnik GmbH confocal microscope. Images were analyzed with the ImageJ software. In order to analyze the cell size, the larval fat bodies were fixed in 4% PFA, permeabilized with 0.2% Triton X-100 in PBS, and rinsed in PBS 1x and membranes were stained with 1 : 100 Alexa Fluor 488 Phalloidin to visualize the cytoskeleton through the binding between Phalloidin and F-actin and Hoechst 33258 for nuclei and then mounted onto slides with DABCO-Mowiol. Photographs were taken using confocal microscopy and the area of adipose cells for each fat body was calculated with ImageJ software. In order to visualize lipids, fat bodies were stained with Nile Red (Sigma Aldrich) and with Alexa Fluor 488 Phalloidin following the protocol in [39].

### 2.3. *In Vivo* Detection of ROS Using Dihydroethidium (DHE)

DHE is used to detect cytosolic superoxides and radical oxygen species (ROS). The reaction between DHE and superoxide anions generates a highly specific red fluorescent product (ethidium), which intercalates with DNA. ROS levels were detected in live tissue as described in [40]. Briefly, larval fat bodies at 5 and 12 days AEL were dissected in Schneider's insect medium (GIBCO). After incubation in 30 *μ*M DHE (Invitrogen) for 5–7 minutes in the dark at room temperature, fat bodies were washed three times with Schneider's medium and immediately mounted with VECTASHIELD Antifade Mounting Medium.

### 2.4. RNA Extraction and Quantitative RT-PCR

Total RNA was extracted from 8 whole larvae using QIAGEN RNeasy Mini Kit. 1 *μ*g total RNA from each genotype was reverse-transcribed into cDNA using SuperScript IV MILO Master Mix (Invitrogen). The obtained CDNA was used as the template for quantitative real-time PCR (qRT-PCR) using SYBR Premix Ex Taq-Tli RnaseH Plus II (TaKara), analyzed on a RT-PCR BIORAD thermocycler machine. In these experiments, gene expression levels were normalized to* actin mRNA*, used as the internal control. The following primers for qRT-PCR were used:* actin5c *5′-CAGATCATGTTCGAGACCTTCAAC-3′ (R) and 5′-ACGACCGGAGGCGTACAG-3′ (F) and E74B 5′-GAATCCGTAGCCTCCGACTGT (R) and 5′-AGGAGGGAGAGTGGTGGTGTT (F) [39].

### 2.5. Protein Extractions and Immunoblotting

The larval fat bodies (10 for each genotype) were dissected in Schneider's medium serum-free and lysed in 80 *μ*l of lysis buffer 1x (50 mM Hepes pH 7.4, 250 mM NaCl, 1 mM EDTA, 1.5% Triton X-100). Protease inhibitor cocktail (Sigma-Aldrich) was added to inhibit protease and phosphatase activities. Samples were sonicated two times for 10 seconds and then centrifuged. Protein concentration was determined by Bradford protein assay (Bio-Rad). The samples were boiled in 1x SDS and then separated on 10% SDS-polyacrylamide gels and blotted. Membrane was incubated with primary antibody anti-phospho-p46 SAPK/JNK (Cell Signaling #9521) or antiactin (Hybridoma Bank) overnight at 4°C in blocking buffer and then washed in 0.1% Tween 20 with TRIS-buffered saline (TBST). Appropriate secondary antibody was incubated for 2 hours, followed by washing. The signal was revealed with ChemiDoc Touch Imaging System (Bio-Rad Lab).

### 2.6. Immunostaining

Dissected fat bodies from 20 larvae were fixed in a solution of 4% PFA/PBS for 40 minutes. After permeabilization with 0.3% Triton/PBS, tissues were washed in a solution of Tween 0.04%/PBS, saturated with 1% BSA/PBS, and incubated overnight with anti-SPARC antibodies (1 : 400), a generous gift from Martinek et al. [41], and visualized using anti-Rabbit Alexa555 (Invitrogen).

### 2.7. Statistical Analysis

The experiments were repeated at least three times and the statistical analysis among the various genotypes was examined by Student's *t*-test. Differences were considered significant if *P values *were less than 0.05 (*∗*), 0.01 (*∗∗*), 0.001 (*∗∗∗*), and 0.0001 (*∗∗∗∗*).

## 3. Results

### 3.1. Obese Larvae Have Increased Size of Fat Cells and Increased Hemocytes in the Fat Body

**Figure 1:**
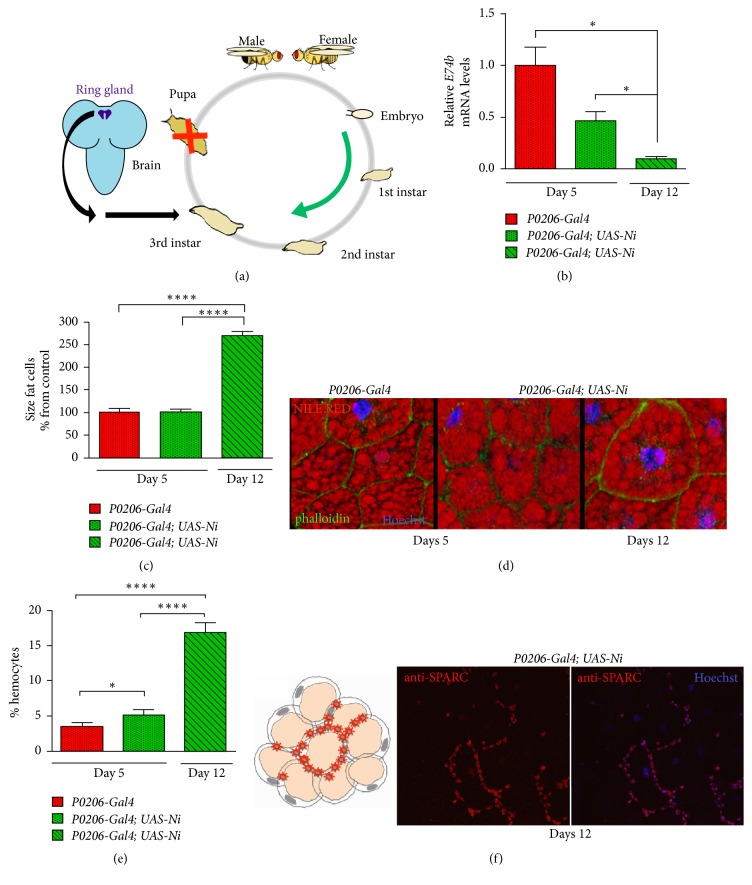
*Obese larvae have increased hemocytes infiltrating the fat cells*. (a) Ecdysone regulation of larval molting and metamorphosis. Reducing the size of the ring gland reduces ecdysone level in* P0206-Gal4; UAS-Ni* animals. (b) Quantitative RT-PCR in whole larvae of the indicated genotype showing the relative expression of* E74b mRNA. Actin5c* was used as control. (c) Relative size of cells from the FBs from animals of the indicated genotypes, at 5 and 12 days AEL. (d) Nile Red staining for lipids, Phalloidin for membranes, and Hoechst for nuclei, of FBs. (e) % of hemocytes infiltrating the FBs of animals at 5 or 12 days AEL, of the indicated genotype. Data are expressed as percentage of hemocytes in the cells of FBs. (f) Draw and confocal photographs of cell from the FB, showing hemocytes stained with anti-SPARC antibodies (red), while nuclei are visualized using Hoechst (blue). Error bars represent SEM (standard error of the mean) of three independent experiments. ^*∗*^*P* < 0,05 and ^*∗∗∗∗*^*P* < 0,0001.

In order to study the ability of hemocytes to infiltrate the fat cells, we blocked pupariation (Figure 1(a)) creating larvae* P0206-Gal4; UAS-Ni* where the reduction of the size of the prothoracic gland, the endocrine organ that produces ecdysone, resulted in reduced levels of ecdysone (Figure 1(b)), leading to animals that develop at almost normal rate and continue to feed until 3 weeks with an increased body weight (see method).* Drosophila* FB-cells function as storage for nutrients, which synthesize and release energy, and accumulate fat and sugars; in our obese animals we observed that at 12 days AEL the size of the cells from the FBs from* P0206-Gal4; UAS-Ni *larvae increased (Figure 1(c)) due also to the accumulation of fats in lipid droplets visible by Nile Red staining (Figure 1(d)). Those* P0206-Gal4; UAS-Ni* animals acquired phenotypic characteristics of obese individuals, including increased triglycerides (TAGs), glucose circulating in the hemolymph, resistance of fat cells to stimulation with insulin, and increased hemocytes in the FB (manuscript in submission).

Chronic inflammation in the adipose tissue is characterized by the infiltration of macrophages in the fat cells; we therefore analyzed if a similar event was present in the FB of our obese animals. We labeled the hemocytes* in vivo* using the* Hml-RFP *reporter line that specifically expresses Red-Fluorescence protein in hemocytes and introduced this transgene to our genetic background.* Hml-RFP* positive cells were monitored over time to visualize and quantify the number of hemocytes infiltrating the FB, from control and obese animals at 5 days AEL and at 12 days AEL in the obese larvae. These results showed that FBs from* P0206-Gal4/Hml-RFP; UAS-Ni *animals contain at 5 days AEL a small but significantly higher number of hemocytes in their FBs (5.2%, *P* < 0.05) as compared to control* P0206-Gal4/Hml-RFP *(Figure 1(e)); furthermore, at 12 days the percentage of hemocytes in* P0206-Gal4/Hml-RFP; UAS-Ni *animals was drastically increased to 17% (*P* < 0.00001). Hemocytes are characterized by the expression of high levels of the cell adhesion protein SPARC (secreted protein acidic and rich in cysteine, also known as osteonectin or BM 40) [41]; morphological analysis of FBs from 12-day* P0206-Gal4; UAS-Ni *animals showed the presence of crown-like structures of hemocytes, positive with anti-SPARC antibodies, that surrounded the fat cells, mimicking similar structures described in the fat of obese individuals suffering from chronic inflammation (Figure 1(f)).

### 3.2. Obese Larvae Have Increased Phosphorylation of JNK/SAPK and of ROS Production in the FB

Chronic inflammation in obese people is often associated with high levels of reactive oxygen species (ROS) and with the activation of the c-Jun-NH_2_-terminal kinase (JNK/SAPK) p46 [15].

**Figure 2:**
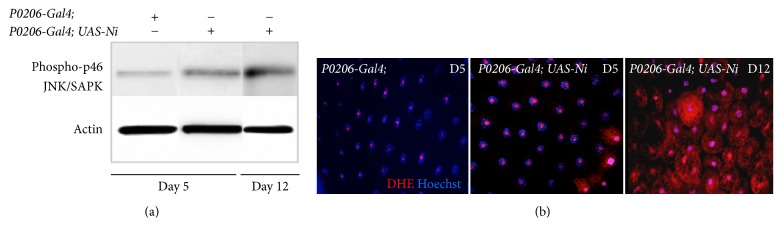
*Obese larvae show activation of JNK/SAPK signaling and increased ROS production*. (a) Western blot from lysates of FBs showing the level of phosphorylation of JNK/SAPN p46 kinase, in* P0206-Gal4* (control) and* P0206-Gal4; UAS-Ni* animals. Actin was used as control loading. (b) Confocal photographs (20x) of cells from FBs stained with DHE (red) for ROS and Hoechst (blue) for nuclei.

We therefore analyzed, in our* Drosophila* model of chronic inflammation, if there was an activation of JNK signaling by looking at the levels of phosphorylation of JNK/SAPK p46. Western blot analysis using extracts from FBs of larvae from* P0206-Gal4 *at 5 days AEL or* P0206-Gal4; UAS-Ni *at 5 and 12 days AEL shows an increase in the phosphorylation of JNK/SAPK p46 kinase (Figure 2(a)).

Since ROS are known to induce the activation of the JNK pathway we then analyzed if in the FBs from the obese larvae there was an increase in ROS signaling, using DHE as a marker. These experiments show that at 5 days AEL, DHE staining increased in FBs from* P0206-Gal4; UAS-Ni *(Figure 2(b), middle panel) animals as compared to control (Figure 2(b), left panel); moreover, DHE staining further increased at 12 days AEL (Figure 2(b), right panel) suggesting that FBs form these animals exhibit significant increase in ROS production over time.

### 3.3. Dietary Anthocyanins Reduce Hemocytes Infiltration in FBs and Phosphorylation of JNKSAPK p46

Flavonoids (FL) and anthocyanins (ACN) are known to have antioxidant effects against inflammation-induced oxidative stress. Therefore, we analyzed if the presence of FL or ACN in the diet of the obese animals had an effect on the chronic inflammation and stress phenotypes.

**Figure 3:**
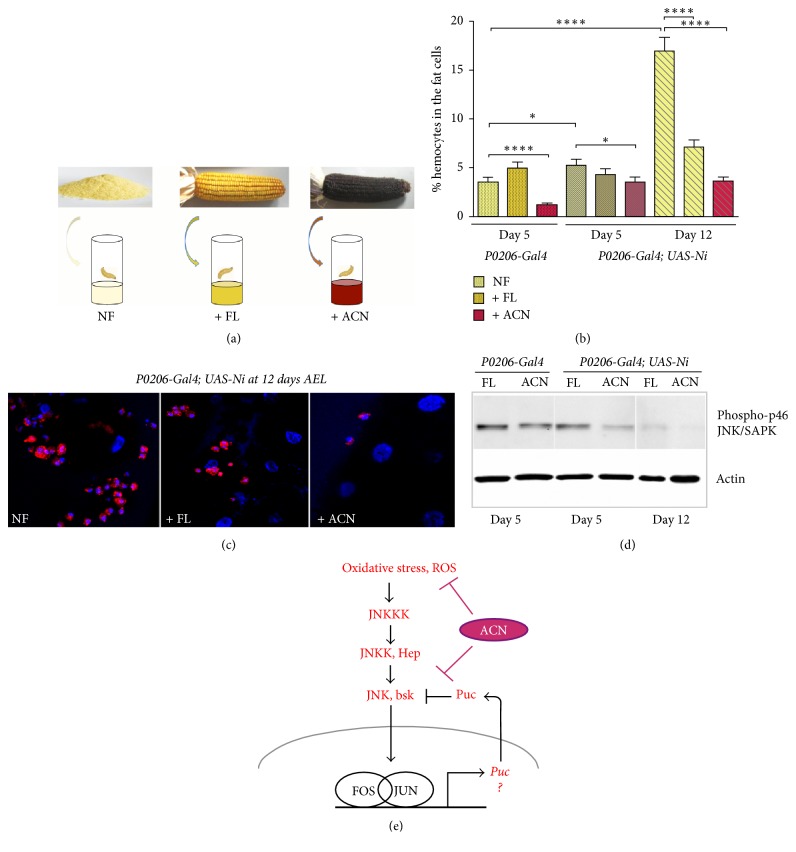
*Anthocyanins-rich diet reduces the infiltration of hemocytes in the FBs and the phosphorylation of JNK/SAPK*. (a) Scheme of the different diets NF (Normal Food) or enriched in FL (flavonoids) or ACN (anthocyanins). (b) % of hemocytes in the cells of the FBs from animals at the indicated genotypes and fed with the indicated diets at 5 or 12 days AEL. (c) Confocal images showing hemocytes expressing Hml-RFP (red) and nuclei stained with Hoechst (blue) from animals at 12 days AEL, upon feeding with NF, FL, or ACN enriched diets. (d) Western blot from lysates of FBs showing the level of phosphorylation of JNK/SAPK p46 kinase, in* P0206-Gal4* (control) and* P0206-Gal4*;* UAS-Ni* animals fed in FL or ACN enriched diets. FBs were taken at 5 or 12 days AEL. Actin was used as control loading. (e) Model of JNK signaling and potential action of anthocianins. Error bars represent SEM (standard error of the mean) of three independent experiments. ^*∗*^*P* < 0,05 and ^*∗∗∗∗*^*P* < 0,0001.

Staged first instar larvae were transferred to normal standard food (NF) or to food enriched with FL only or enriched with FL + ACN, herein called ACN (Figure 3(a) and material and methods), and their effect on the migration of hemocytes in the FBs was quantified by visualizing the number of HML-RFP positive cells on dissected FBs using a fluorescent microscope. These experiments show that after 5 days of feeding with the different diets, only food containing ACN significantly reduces from 5.7% to 3.2% the presence of hemocytes in the FBs of* P0206-Gal4; UAS-Ni* animals, while treatment with FL did not have any effect (Figure 3(b)). At 12 days instead both FL and ACN diets were able to significantly decrease the number of hemocytes (Figure 3(b)). In addition macroscopic analysis of the shape and number of hemocytes showed that at 12 days AEL both FL and ACN treatments were able to reduce the formation of crown-like structures of hemocytes surrounding the fat cells in* P0206-Gal4; UAS-Ni* animals (middle and left panel) Figure 3(c). We then analyzed the effect of FL and ACN diets on the phosphorylation of the stress-response JNK/SAPK p46; FBs from animals growing in the different diets were dissected at 5 and 12 days AEL and phosphorylation of JNK/SAPK was analyzed by western blot. These experiments showed that at 5 days AEL, feeding with ACN significantly reduced the phosphorylation of JNK/SAPK p46 in FBs from* P0206-Gal4; UAS-Ni* (Figure 3(d)), while after 12 days AEL both diets with FL and ACN were able to significantly reduce JNK/SAPK p46 phosphorylation, suggesting a potential role at later points for FL in reducing oxidative stress.

## 4. Discussion

Obesity and metabolic disorders are pathological conditions associated with our diet enriched of fats and sugars or with a sedentary life, but also with environmental factors that may pollute our food with chemicals that affect lipid metabolism. As consequences we have seen an increase in cardiovascular diseases, type 2 diabetes, and a chronic inflammation of the adipose tissue (ATM) induced by the persistent infiltration of macrophages into the fat cells, for which the mechanisms are not totally understood but have been associated with an oxidative stress condition present between the immune cells in the metabolic tissues. In order to study* in vivo* these relationship, we have taken advantage of the conserved relationship in* Drosophila* between the immune cells (hemocytes) and the fat body (adipose tissue) to study how bioproducts like flavonoids in particular anthocyanins that are known to act as antioxidants and that are naturally present in our food may ameliorate or counteract the migration of the hemocytes into the FB using our animal model that mimics chronic inflammation in vertebrate (ATM).

Anthocyanins are a class of flavonoids classified as bioactive food have been shown to ameliorate hyperglycemia, insulin sensitivity, and fat accumulation in obese mice fed to a high fat diet and in vertebrates studies identify a beneficial effect by anthocyanins in combating inflammation-related diseases such as diabetes, cardiovascular diseases, and obesity [25, 42, 43]. Moreover, clinical studies in humans demonstrate that higher consumption of anthocyanins is associated with weight loss in both men and women and reduces the risk of developing chronic diseases with a mechanism poorly understood [24, 44].

In this study, we are using our innovative model that mimic obesity in flies, where upon blocking growth by reducing ecdysone, the animals develop at almost normal rate but continue to feed with an increase in body weight and in the fat cell-size; these animals acquire the characteristics of obese people, with an accumulation of TAGs and insulin resistance (manuscript in preparation). Moreover, these animals present an infiltration of hemocytes (macrophage-like cells) within the cells of the FB that progressively increases until the formation of the typical “crown-like structures” described in obese patients suffering from ATM [5]. We demonstrate that in FBs from these obese animals there is increased production of ROS, indicating the presence of an oxidative stress that may be responsible of the augmented phosphorylation of the JNK/SAPK p46 stress kinase. Because the molecular mechanisms that regulate lipid metabolism are highly conserved between humans and flies [45, 46] and hemocytes have been shown to be functionally equivalent to macrophages we can speculate that the mechanisms underlining these humoral responses are conserved also in flies. Therefore, we use our obesity model to investigate the antioxidant effect of flavonoids and anthocyanins to chronic inflammation. In our study, we find that a diet rich in anthocyanins reduces hemocytes migration in the larval FB and decreases the accumulation of TAGs in the fat cells (not shown) ameliorating several characteristics of the obese phenotypes. Moreover, we showed that anthocyanins reduce the production of ROS in cells of the FB and significantly attenuate the phosphorylation of JNK/SAPK p46 kinase providing evidence that may play a key role in regulating the JNK-mediated cellular stress responses and to control ROS signaling.

The interplay of signals that regulate the nonautonomous responses between hemocytes and the cells of the FB is coming up as a new field for important studies; indeed recently hemocytes have been shown to be responsible of mediating an humoral immune response in a model for tumor growth, where they were shown to trigger signals responsible of killing the tumor cells through a nonautonomous mechanism mediated by the activation of cytokines of the Toll and Eiger/TNF*α* by the fat body [34]. More recently, hemocytes were shown that upon stress conditions they are able to migrate near epithelial cells and to produce ROS to induced the release of Eiger/TNF*α* by the epithelial cells through the activation of the JNK signaling pathway, suggesting also in this case the presence of nonautonomous signals between the hemocytes and the cell of the epithelium necessary for tissue homeostasis [35, 47, 48]. In a similar way, we can speculate that the hemocytes in the FB from obese animals may be activated by the oxidative stress signals (ROS), present in the FB, that trigger signals to induce the production of cytokines of the Toll and Eiger/TNF*α* that further aggravate the oxidative stress condition that attract the hemocytes that constitutively migrate into the fat cells causing a status of chronic inflammation.

In our experiments, we show that anthocyanins are able to reduce the activation of JNK/SAPK p46 stress kinase. As mentioned before, JNK pathway is activated upstream by ROS and by cytokines including Eiger/TNF*α*; this pathway is inhibited by a negative regulatory feedback that induces the transcription of the phosphatase* puckered* (Figure 3(e)). In our model, we can speculate that anthocyanins may either directly block cytokines upstream of JNK signal, for example, by controlling Eiger/TNF*α* signaling, or they may contribute to the activation of the negative feedback that involved the activity of puckered.

Interestingly, anthocyanins were shown to act concomitantly with detoxification enzymes such as superoxide dismutase, catalase, glutathione peroxidase, glutathione-S-transferase (GST), and glutathione reductase to reduce oxidation. In* Drosophila Gst-D1* [49] and* jafrac,* an inhibitor of cell death, together with* puckered*, were shown to be transcriptional targets of* jun-fos* activity in response to the activation of JNK pathway, and these genes were shown to negatively counteract the oxidative stress response [50]. Our preliminary data however did not find any regulation in the expression of* GstD1* in the fat cells from the obese animals upon feeding with FL or ACN anthocyanins (data not shown) suggesting that probably this enzyme is not involved in the regulation of JNK signaling by anthocyanins in these cells.

In conclusion, with the present study we provide for the first time a strong evidence of the potential use of anthocyanins in the diet to control chronic inflammation and provide a link to the oxidative stress that characterize the adipose tissue in obese animals. We were able to evidence the ability of anthocyanins to decrease* in vivo* the phosphorylation of JNK/SAPK p46 stress kinase, thus providing a new insight into the mechanism of phenolic compounds in the treatment of inflammation in adipose tissues, a field of currently study since the lack of a better knowledge of the mechanisms that regulate or control ATM in pathologies such as obesity and metabolic disorders.

## References

[1] Chung W., Park C. G., Ryu O.-H. (2016). Association of a new measure of obesity with hypertension and health-related quality of life. *PLoS ONE*.

[2] Yang C., Kong A. P., Cai Z., Chung A. C. (2017). Persistent Organic Pollutants as Risk Factors for Obesity and Diabetes. *Current Diabetes Reports*.

[3] Karnik S., Kanekar A. (2012). Childhood obesity: A global public health crisis. *International Journal of Preventive Medicine*.

[4] Kaila B., Raman M. (2008). Obesity: A review of pathogenesis and management strategies. *Canadian Journal of Gastroenterology & Hepatology*.

[5] Wellen K. E., Hotamisligil G. S. (2003). Obesity-induced inflammatory changes in adipose tissue. *The Journal of Clinical Investigation*.

[6] Mraz M., Haluzik M. (2014). The role of adipose tissue immune cells in obesity and low-grade inflammation. *Journal of Endocrinology*.

[7] Gregor M. F., Hotamisligil G. S. (2011). Inflammatory mechanisms in obesity. *Annual Review of Immunology*.

[8] Lee B.-C., Lee J. (2014). Cellular and molecular players in adipose tissue inflammation in the development of obesity-induced insulin resistance. *Biochimica et Biophysica Acta (BBA) - Molecular Basis of Disease*.

[9] Newton K., Dixit V. M. (2012). Signaling in innate immunity and inflammation. *Cold Spring Harbor Perspectives in Biology*.

[10] Horng T., Hotamisligil G. S. (2011). Linking the inflammasome to obesity-related disease. *Nature Medicine*.

[11] Arango Duque G., Descoteaux A. (2014). Macrophage cytokines: involvement in immunity and infectious diseases. *Frontiers in Immunology*.

[12] Scheller J., Chalaris A., Schmidt-Arras D., Rose-John S. (2011). The pro- and anti-inflammatory properties of the cytokine interleukin-6. *Biochimica et Biophysica Acta (BBA) - Molecular Cell Research*.

[13] Rani V., Deep G., Singh R. K., Palle K., Yadav U. C. S. (2016). Oxidative stress and metabolic disorders: pathogenesis and therapeutic strategies. *Life Sciences*.

[14] Matsuda M., Shimomura I. (2013). Increased oxidative stress in obesity: implications for metabolic syndrome, diabetes, hypertension, dyslipidemia, atherosclerosis, and cancer. *Obesity Research & Clinical Practice*.

[15] Kyriakis J. M., Banerjee P., Nikolakaki E. (1994). The stress-activated protein kinase subfamily of c-jun kinases. *Nature*.

[16] Martín-Blanco E., Gampel A., Ring J. (1998). Puckered encodes a phosphatase that mediates a feedback loop regulating JNK activity during dorsal closure in *Drosophila*. *Genes & Development*.

[17] Engin A. B. (2017). What is lipotoxicity?. *Advances in Experimental Medicine and Biology*.

[18] Rosca M. G., Vazquez E. J., Chen Q., Kerner J., Kern T. S., Hoppel C. L. (2012). Oxidation of fatty acids is the source of increased mitochondrial reactive oxygen species production in kidney cortical tubules in early diabetes. *Diabetes*.

[19] Boden G. (2008). Obesity and free fatty acids. *Endocrinology and Metabolism Clinics of North America*.

[20] Petroni K., Pilu R., Tonelli C. (2014). Anthocyanins in corn: a wealth of genes for human health. *Planta*.

[21] Lee Y.-M., Yoon Y., Yoon H., Park H.-M., Song S., Yeum K.-J. (2017). Dietary anthocyanins against obesity and inflammation. *Nutrients*.

[22] Sotibran A. N. C., Ordaz-Tellez M. G., Rodriguez-Arnaiz R. (2011). Flavonoids and oxidative stress in Drosophila melanogaster. *Mutation Research - Genetic Toxicology and Environmental Mutagenesis*.

[23] Prochazkova D., Bousova I., Wilhelmova N. (2011). Antioxidant and prooxidant properties of flavonoids. *Fitoterapia*.

[24] Bertoia M. L., Rimm E. B., Mukamal K. J., Hu F. B., Willett W. C., Cassidy A. (2016). Dietary flavonoid intake and weight maintenance: Three prospective cohorts of 124 086 US men and women followed for up to 24 years. *BMJ*.

[25] Tsuda T., Horio F., Uchida K., Aoki H., Osawa T. (2003). Dietary cyanidin 3-O-*β*-D-glucoside-rich purple corn color prevents obesity and ameliorates hyperglycemia in mice. *Journal of Nutrition*.

[26] Zheng H., Yang X., Xi Y. (2016). Fat body remodeling and homeostasis control in Drosophila. *Life Sciences*.

[27] Liu Y., Liu H., Liu S., Wang S., Jiang R.-J., Li S. (2009). Hormonal and nutritional regulation of insect fat body development and function. *Archives of Insect Biochemistry and Physiology*.

[28] Buchon N., Silverman N., Cherry S. (2014). Immunity in *Drosophila melanogaster*—from microbial recognition to whole-organism physiology. *Nature Reviews Immunology*.

[29] Ganesan S., Aggarwal K., Paquette N., Silverman N. (2011). Nf-*κ*B/Rel proteins and the humoral immune responses of *Drosophila melanogaster*. *Current Topics in Microbiology and Immunology*.

[30] Leulier F., Lemaitre B. (2008). Toll-like receptors - Taking an evolutionary approach. *Nature Reviews Genetics*.

[31] Tokusumi Y., Tokusumi T., Shoue D. A., Schulz R. A. (2012). Gene regulatory networks controlling hematopoietic progenitor niche cell production and differentiation in the Drosophila lymph gland. *PLoS ONE*.

[32] Lemaitre B., Hoffmann J. (2007). The host defense of *Drosophila melanogaster*. *Annual Review of Immunology*.

[33] Evans C. J., Hartenstein V., Banerjee U. (2003). Thicker than blood: Conserved mechanisms in Drosophila and vertebrate hematopoiesis. *Developmental Cell*.

[34] Parisi F., Stefanatos R. K., Strathdee K., Yu Y., Vidal M. (2014). Transformed epithelia trigger non-tissue-autonomous tumor suppressor response by adipocytes via activation of toll and eiger/TNF signaling. *Cell Reports*.

[35] Fogarty C. E., Diwanji N., Lindblad J. L. (2016). Extracellular Reactive Oxygen Species Drive Apoptosis-Induced Proliferation via Drosophila Macrophages. *Current Biology*.

[36] Colombani J., Bianchini L., Layalle S. (2005). Antagonistic actions of ecdysone and insulins determine final size in Drosophila. *Science*.

[37] Pilu R., Cassani E., Sirizzotti A., Petroni K., Tonelli C. (2011). Effect of flavonoid pigments on the accumulation of fumonisin B1 in the maize kernel. *Journal of Applied Genetics*.

[38] Makhijani K., Alexander B., Tanaka T., Rulifson E., Brückner K. (2011). The peripheral nervous system supports blood cell homing and survival in the Drosophila larva. *Development*.

[39] Parisi F., Riccardo S., Zola S. (2013). DMyc expression in the fat body affects DILP2 release and increases the expression of the fat desaturase Desat1 resulting in organismal growth. *Developmental Biology*.

[40] Owusu-Ansah E., Banerjee U. (2009). Reactive oxygen species prime *Drosophila* haematopoietic progenitors for differentiation. *Nature*.

[41] Martinek N., Zou R., Berg M., Sodek J., Ringuette M. (2002). Evolutionary conservation and association of SPARC with the basal lamina in Drosophila. *Development Genes and Evolution*.

[42] Xie B., Waters M. J., Schirra H. J. (2012). Investigating potential mechanisms of obesity by metabolomics. *Journal of Biomedicine and Biotechnology*.

[43] Azzini E., Giacometti J., Russo G. L. (2017). Antiobesity Effects of Anthocyanins in Preclinical and Clinical Studies. *Oxidative Medicine and Cellular Longevity*.

[44] Guo H., Ling W. (2015). The update of anthocyanins on obesity and type 2 diabetes: Experimental evidence and clinical perspectives. *Reviews in Endocrine and Metabolic Disorders*.

[45] Hirabayashi S. (2016). The interplay between obesity and cancer: A fly view. *Disease Models & Mechanisms*.

[46] Trinh I., Boulianne G. L. (2013). Modeling obesity and its associated disorders in Drosophila. *Physiology Journal*.

[47] Igaki T., Miura M. (2014). The *Drosophila* TNF ortholog Eiger: emerging physiological roles and evolution of the TNF system. *Seminars in Immunology*.

[48] Wang M. C., Bohmann D., Jasper H. (2003). JNK signaling confers tolerance to oxidative stress and extends lifespan in *Drosophila*. *Developmental Cell*.

[49] Udomsinprasert R., Bogoyevitch M. A., Ketterman A. J. (2004). Reciprocal regulation of glutathione S-transferase spliceforms and the Drosophila c-Jun N-terminal kinase pathway components. *Biochemical Journal*.

[50] Khoshnood B., Dacklin I., Grabbe C. (2016). Urm1: An essential regulator of JNK signaling and oxidative stress in Drosophila melanogaster. *Cellular and Molecular Life Sciences*.

